# Development of Malaysian-MIND diet scores for prediction of mild cognitive impairment among older adults in Malaysia

**DOI:** 10.1186/s12877-024-04966-7

**Published:** 2024-05-01

**Authors:** Muhamad Mustaqim M Zapawi, Yee Xing You, Suzana Shahar, Mohd Razif Shahril, Nurul Fatin Malek Rivan, Nik Nur Izzati Nik Mohd Fakhruddin, Anastasia Xin Wei Yap

**Affiliations:** 1 Dietetic Programme, Centre for Healthy Ageing and Wellness (HCARE), Faculty of Health Sciences, Universiti Kebangsaan Malaysia, Jalan Raja Muda Abdul Aziz, Kuala Lumpur, 50300 Malaysia; 2 Nutrition Programme, Centre for Healthy Ageing and Wellness (HCARE), Faculty of Health Sciences, Universiti Kebangsaan Malaysia, Jalan Raja Muda Abdul Aziz, Kuala Lumpur, 50300 Malaysia; 3 Jeffrey Cheah School of Medicine and Health Sciences, Monash University Malaysia, Jalan Lagoon Selatan, Bandar Sunway, Selangor Darul Ehsan 47500 Malaysia

**Keywords:** Malaysian-MIND diet, Dietary pattern, Prediction, Mild cognitive impairment, Older adult

## Abstract

**Background:**

Mild Cognitive impairment (MCI) is a pre-demented state in the elderly populace. The Mediterranean & Dietary Approaches to Stop Hypertension (DASH) Intervention for Neurodegenerative Delay (MIND) diet has shown promise in reducing the risk of MCI and Alzheimer’s disease in older people. Notably, the existing MIND diet is not adapted to the specific needs of older adults in Malaysia, considering distinct food cultures and availability. Consequently, this study aimed to develop the Malaysian version of the MIND diet (MY-MINDD) scores and investigate their association with MCI in the older adult populace of Malaysia.

**Methods:**

A comprehensive pooled data analysis was conducted on combined data from 810 participants sourced from the longitudinal Long-Term Research Grant Scheme-Towards Useful Aging (LRGS-TUA) and Fundamental Research Grant Scheme (FRGS) studies. The MY-MINDD scores were developed by incorporating existing MIND diet food groups, their corresponding scoring mechanisms, and consideration of common Malaysian foods which are proven to be beneficial and detrimental to cognitive function. To substantiate the MY-MINDD scoring system, its association with MCI was evaluated using a series of validated neuropsychological test batteries.

**Results:**

MY-MINDD consists of seven food groups promote brain health and four food groups exert negative cognitive outcomes. The study participants had an average age of 67.9 ± 4.7 years. The collective MY-MINDD score for all participants was 6.4 ± 0.1 (out of a maximum 11 points), revealing a lower score in individuals with MCI at 6.0 ± 1.7 compared to those without MCI at 6.6 ± 1.6 (*p* < 0.001). According to hierarchical multivariate binary logistic regression analysis, being in the highest tertile of MY-MINDD score was linked to reduced odds of MCI (odds ratio (OR) = 0.43, 95% confidence interval (CI): 0.26–0.72, *p* < 0.001) in the fully adjusted model in comparison to the lowest tertile.

**Conclusion:**

The development of the MY-MINDD scores for Malaysian older population revealed that a stronger adherence to this diet is linked to a reduced risk of MCI. Further substantiation of the MY-MINDD scores using more objective measures, such as neuroimaging approaches and other neuropsychological batteries, is necessary.

## Background

The global populace is rapidly aging, with the World Health Organization (2022) projecting that by 2030, one in six individuals will be 60 years or older, reaching a population of 2.1 billion by 2050 [1]. Aging is closely linked with neurodegenerative ailments, particularly irreversible dementia, with a notable prevalence in rapidly emerging Asian nations like Malaysia [2]. The prevalence of mild cognitive impairment (MCI), the pre-demented state varies based on geographical location and criteria utilised for its definition. Across different regions, rates range from 14.9% in India to 18.5% in China and 20.0% in Luxemburg [3]. As of now, the prevalence of dementia and MCI among individuals aged 60 and above in Malaysia is reported at 8.5% and 16%, respectively [3, 4].

As individuals age, alterations in the brain become evident due to homeostatic dysregulation, characterised by hormonal shifts and damage from oxidative stress [5]. These changes contribute to neurodegeneration or neuroplasticity, leading to phenomena like brain atrophy and neuroinflammation, ultimately resulting in cognitive decline as part of the aging process [6]. Both unmodifiable (such as aging and female sex) and modifiable (like smoking, obesity, diet, hypertension, diabetes, and heart disease) can increase the risk of cognitive impairment [7]. Within modifiable lifestyle factors, dietary choices have been linked to cognitive decline and Alzheimer’s disease. Existing literature demonstrates that the Mediterranean & Dietary Approaches to Stop Hypertension (DASH) Intervention for Neurodegenerative Delay (MIND) diet, an amalgamation of the Mediterranean diet and the DASH diet, initially devised by Morris et al. (2015), effectively mitigates cognitive decline in older individuals and decreases the risk of Alzheimer’s disease [8]. The MIND diet has ample rich in nutrients like vitamin E, folate, lutein-zeaxanthin, and flavonoids, which are antioxidant, anti-inflammatory, and able to enhance cognition properties [8, 9]. Various versions of the MIND diet are implemented globally, including the original from the United States [10], the Japanese-MIND diet in Japan [11], and the cMIND diet in China [7]. Notably, the current iterations of the MIND diet feature distinct food groups, diverging from those suitable for Southeast Asian nations like Malaysia, given their unique food cultures and availability [12].

As per a comprehensive review by Kheirouri and Alizadeh (2021), encompassing 13 studies, the MIND diet exhibited a positive association with global cognitive function and outperformed other diets rich in plant-based components [13]. In Japan, participants rigorously adhering to the MIND diet experienced a 50% reduction in Alzheimer’s disease incidence compared to those not following it [11]. While previous studies primarily explored the link between MIND diet adherence and the decreased risk of cognitive decline among older individuals in Western nations and two Asian nations [8], no research has investigated the association between adherence to the MIND diet and enhanced cognitive function, particularly in Southeast Asian nations. Therefore, the objective of this study was aimed to develop a Malaysian adaptation of the MIND diet, along with a scoring system, and assess the association between these scores and MCI among older adults in Malaysia.

## Methods

### Study design

This study is a comprehensive pooled data analysis on combined data from 810 participants sourced from the longitudinal Long-Term Research Grant Scheme-Towards Useful Aging (LRGS-TUA) [14] and Fundamental Research Grant Scheme (FRGS) [15] studies to develop the Malaysian-MIND diet.

### Development of Malaysian-MIND diet

**Fig. 1:**
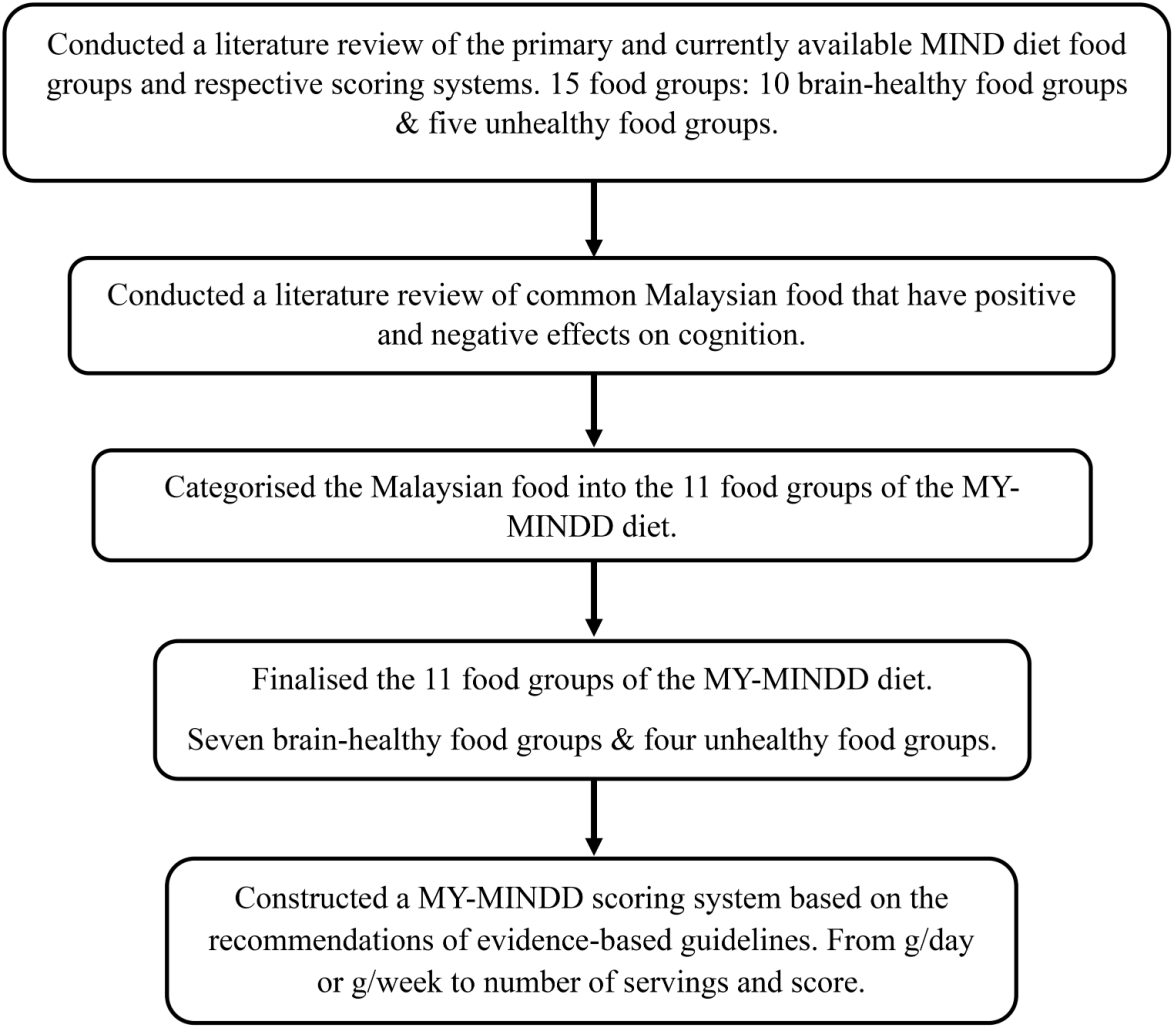
A flowchart depicting the process of developing the MY-MINDD diet

Illustrated in Fig. 1, the development of the Malaysian version of MIND diet (MY-MINDD) scoring system involved a series of steps. It commenced with a thorough review of literature on both the primary MIND diet and existing food groups, coupled with their corresponding scoring methodologies [10]. Additionally, an exploration of common Malaysian foods which exert positive and negative cognitive effects was conducted. The food items were then classified on the basis of 11 food groups in MY-MINDD. A total of 11 food groups comprising seven brain-healthy food groups and four unhealthy food groups were created after discussions among research team members. The research team comprised of four dietitians and two nutritionists. To quantify the intake, each food item was converted into serving sizes using the Nutritionist Pro (Axxya Systems Stafford, USA) software, the Atlas of Food Exchanges & Portion Sizes UKM [16], and the Malaysian Food Composition database [17]. The construction of MY-MINDD scores was then based on the recommended serving sizes for every food group as per evidence-based guidelines in Malaysia [12, 18, 19].

Table 1 provides a comparison of dietary components and scores between the original MIND diet and MY-MINDD. The primary MIND diet incorporated 10 brain-healthy food groups, encompassing green leafy vegetables, other vegetables, berries, nuts, beans, not-fried fish, not-fried poultry, whole grains, olive oil, and wine. Additionally, it included five unhealthy groups: red meats, butter and stick margarine, cheese, pastries and sweets, and fried/fast food [8].

**Table 1:** Comparison of the dietary components and scores between the original MIND diet and the Malaysian-MIND diet (MY-MINDD)

MIND diet				Malaysian-MIND diet (MY-MINDD)			
Components		**Score**		**Components**		**Score**	
	**0**	**0.5**	**1**		**0**	**0.5**	**1**
Whole grains	< 1 serving/ day	1–2 servings/ day	≥ 3 servings/ day	Whole grains	< 1 serving/ day	1 to < 3 servings/day	≥ 3 servings/ day
Green leafy vegetables	≤ 2 servings/ week	> 2 to < 6 servings/week	6 servings/ week	Green leafy vegetables	< 0.5 serving/ day	0.5 to < 1 serving/day	≥ 1 serving/ day
Other vegetables	< 5 servings/ week	5 to < 7 servings/week	≥ 1 serving/ day	Other vegetables	< 1 serving/ day	1 to < 3 servings/day	≥ 3 servings/ day
Berries	< 1 serving/ week	1 serving/ week	≥ 2 servings/ week	Flavonoid-rich fruits	< 1 serving/ week	1 serving/ week	≥ 2 servings/ week
Olive oil	Not primary oil		Primary oil used				
Fish (not fried)	Rarely	1–3 servings/ month	≥ 1 meals/ week	Deep-sea fish (not fried)	< 0.5 serving/ week	0.5 to < 1 serving/week	≥ 1 serving/ week
Beans	< 1 meal/ week	1–3 servings/ week	> 3 meals/ week	Legumes and Soy Products	< 1 serving/ week	1 to < 3 servings/ week	≥ 3 servings/ week
Nuts	< 1 serving/ month	1 serving/ month - <5 servings/week	≥ 5 servings/ week				
Poultry (not fried)	< 1 meal/ week	1 serving/ week	≥ 2 meals/ week	Poultry (not fried)	< 1 serving/ week	1 serving/ week	≥ 2 servings/ week
Wine	> 1 glass/day or never	1 serving/ month − 6 servings/week	1 glass/ day				
Pastries and sweets	≥ 7 servings/ week	5–6 servings/ week	< 5 servings/ week	Desserts, Sweetened *Kuih* & Beverages	≥ 7 servings/ week	5 to 6 servings/week	< 5 servings/ week
Butter, margarine	> 2 servings/ day	1–2 servings/ day	< 1 serving/day	Butter, Margarine	> 2 Tbsp/ day	1 to 2 Tbsp/ day	< 1 Tbsp/ day
Cheese	≥ 7 servings/ week	1–6 servings/ week	< 1 serving/week				
Red meat and products	≥ 7 meals/ week	4–6 servings/ week	< 4 meals/ week	Red meat	≥ 7 servings/ week	4–6 servings/ week	< 4 servings/ week
Fried/fast foods	≥ 4 times/ week	1–3 servings/ week	< 1 time/ week	Fried/fast foods	≥ 4 servings/ week	1–3 servings/ week	< 1 serving/ week

*Abbreviations* MIND, Mediterranean-DASH Intervention for Neurodegenerative Delay; MY-MINDD, Malaysian version of the MIND

In a recent study, there was a relationship between increased whole grain consumption and a decelerated rate of cognitive decline in African American participants, impacting global cognition, episodic memory, and perceptual speed hence the whole grains group was listed as one of the important component in the MY-MINDD [20]. Green leafy vegetables serve as rich sources of vitamin C, folate, carotenes, and flavonoids, contributing to a slower cognitive decline and a reduced incidence of dementia [7]. Fruits and vegetables contain a significant amount of minerals, vitamins and polyphenols that provide protection against free radicals [21]. In the MY-MINDD, we advocate for the consumption of high flavonoid fruits, green leafy and other vegetables. As per the Malaysian Dietary Guidelines (2020), the recommended daily intake for fruits is two servings; for vegetables, it is ≥ three servings. Previous research has highlighted a positive correlation between consuming high amounts of fruits and vegetables (up to 500 g/day) and improved cognitive performance [22]. Recent epidemiological observations have linked the consumption of anthocyanin-rich foods, like berries, to a reduced risk of cognitive impairment [22]. Notably, the limited affordability and availability of berries in Malaysia have hindered their widespread consumption [23]. Therefore, we have replaced the berries group in the MY-MINDD diet with flavonoid-rich fruits like dried prune, roselle, raisin, pomegranate, red dragon fruit, and pink guavas, all of which are expected to exert positive cognitive impacts [24]. Due to the relatively high cost and limited usage of olive oil in Malaysia, we have opted to exclude it from the MY-MINDD diet [25].

Deep-sea fish serves as a rich reservoir of n-3 polyunsaturated fatty acids renowned for their anti-inflammatory properties [26]. The omega-3 fatty acids derived from deep-sea fish play a crucial role in neuronal membranes, and existing literature indicates that increased omega-3 intake is linked to reduced brain atrophy and cognitive decline [7]. Epidemiologic studies have demonstrated an inverse association between omega-3 fatty acids and the onset of cognitive impairment and dementia [27, 28], indicating a protective role in averting neuronal damage and fostering cognitive improvement [29]. Malaysia boasts several omega-3-rich deep-sea fish varieties, including sardines (*sardine*), mackerel (*kembong*), king mackerel (*tenggiri*), torpedo scad (*cencaru*), yellowstripe scad (*selar kuning*), and threadfins (*senangin*) [30, 31], all of which are incorporated into the food groups of MY-MINDD. Frying is a cooking method that significantly diminishes the content of n-3 and n-6 fatty acids [32]. Prolonged exposure to acrylamide, a common contaminant in fried products, promotes lipid peroxidation and oxidative stress, contributing to cerebral neuroinflammation [33]. Therefore, the MY-MINDD diet encourages the consumption of not-fried poultry and deep-sea fish to support cognitive health.

Legumes and soy products substituted beans and nuts, given their limited consumption among the Malaysian populace. Soybeans encompass various components, including soy protein and non-protein soy elements like isoflavones, which offer numerous physiological benefits such as antihypertensive, hypolipidemic, anti-inflammatory, antioxidant properties, and better glycaemic control [34, 35]. Legumes are rich in bioactive compounds with anti-inflammatory effects, crucial for mitigating inflammation and oxidative stress, and they are a valuable source of minerals, protein, vitamin B, and beneficial phytochemicals with biological effects [36]. A prior study has advocated increasing legume intake to three servings per week for a protective impact against cognitive decline [37].

Wine and cheese were excluded from MY-MINDD as they are infrequently consumed among older adults in Malaysia. In lieu of pastries, sweets, and fried/fast foods, these were replaced with desserts, sweetened *kuih*, beverages, and fried/fast foods, which are more familiar to the Malaysian populace. However, the discouraged food components in the MY-MINDD, such as fried/fast foods including banana fritters, banana ball, *cucur*, curry puff, and *cakoi*, could elevate the risk of inflammation-induced ailments like high blood pressure and atherosclerosis, which are linked to cognitive decline and dementia [38, 39]. Additionally, such fried foods are frequently fried using reused cooking oil, impeding the activity of paraoxonase enzyme and leading to an accumulation of low-density lipoprotein (LDL) cholesterol, contributing to the development of cognitive decline and atherosclerosis [40]. Excessive consumption of sugar/sweets from desserts, sweetened *kuih*, and beverages can disrupt insulin and glucose metabolism, and raise neuroinflammation and oxidative stress, ultimately causing structural changes in the normal brain [7]. MY-MINDD also discourages the intake of red meat, butter and margarine, as many prospective research works have indicated a correlation between a diet low in saturated and trans-unsaturated (hydrogenated) fats and slower incidences of cognitive decline [41–43].

The 11 food groups (whole grains, green leafy vegetables, other vegetables, flavonoid-rich fruits, deep-sea fish (not fried), poultry (not fried), legumes and soy products, desserts, sweetened *kuih* and beverages, red meat, fried/fast foods and butter/margarine) were assigned scores on the basis of consumption frequency, with values of 0, 0.5, and 1 corresponding to the respective serving sizes. The total score was computed by summing up all individual component scores, resulting in a maximum score of 11, which is lower than the actual version with a total score of 15 [8]. A higher score reflects a stronger adherence to MY-MINDD.

### Validation with pooled data

This constitutes a pooled data analysis involving a total of 810 participants drawn from the Long-Term Research Grant Scheme-Towards Useful Aging (LRGS-TUA) longitudinal study and the Fundamental Research Grant Scheme (FRGS) study. Initially, there were 2210 raw data entries from LRGS-TUA, but only 579 were chosen following a comprehensive food group analysis [14]. Subsequently, an additional 231 data points from the FRGS study were incorporated into the analysis [15]. The parameters derived from these studies were employed for validation against MCI, as depicted in Table 2.

**Table 2:** Validation parameters against MCI

Parameters	Tools/Methods
Sociodemographic, health, lifestyle	Standard questionnaire [44]
Anthropometry data	Weight, height, BMI [44]
Dietary assessment	Dietary history questionnaire (DHQ) [45]
Depressive symptoms	Geriatric Depression Scale-15 (GDS-15) [46]
Functional status	Instrumental Activities of Daily Living (IADL) [47]
Cognitive function status	Neuropsychological test batteries: Malay version Mini-Mental State Examination (M-MMSE) [48], Digit span test [49], Rey Auditory Verbal Learning Test (RAVLT) [50], Digit symbol substitution [49], Visual reproduction (VR) test [51]

### Mild cognitive impairment

Mild cognitive impairment (MCI) was categorised in accordance with the criteria established by Peterson et al. [52] and Lee et al. [53], encompassing preserved global function, objective memory impairment (a minimum of 1.5 standard deviations below the mean for Rey Auditory Verbal Learning Test (RAVLT)), absence of limitations in instrumental activities of daily living (IADL), subjective memory complaints, and confirmation of no dementia by an accredited medical officer.

### Covariates

Covariates encompassed sociodemographic and lifestyle factors, health conditions and psychosocial aspects, as well as nutritional and dietary considerations. Sociodemographic and lifestyle variables included age, education years, household income and smoking status, with smoking history classified as “no” (never smoked) or “yes” (previously or currently smoked). Health conditions and psychosocial aspects were self-reported and covered functional status, depressive symptoms, hypertension and diabetes. Depressive symptoms were evaluated using GDS-15 [46], while functional status was evaluated through IADL [47]. Anthropometric parameters, specifically body mass index (BMI), were chosen as covariates. BMI, derived from measured weight and height, was classified into four groups: underweight (< 18.5), normal (18.5–24.9), overweight (25.0–29.9), and obese (≥ 30) [54]. Diagnosis of diabetes and hypertension relied on self-reports confirmed by a medical doctor [19].

### Statistical analysis

The statistical analysis was conducted using IBM SPSS for Windows version 28.0 software, having a significance level set at *p* < 0.05. Descriptive data were employed to present the frequency and percentage of baseline attributes for the participants. Sociodemographic data, tested for normality using the Kolmogorov-Smirnov test (*p* > 0.05), were presented as number percentages or mean and standard deviation. To identify significant differences between continuous variables and each tertile of the diet score, a one-way ANOVA test was applied. To identify significant differences between continuous variables of MCI and non-MCI, Mann-Whitney test was applied. The Crosstab Chi-square test was utilised for categorical variables. The Pearson Correlation test was used to determine the association between the MY-MINDD score with cognitive test by obtaining the value of correlation coefficient (*r*).

Hierarchical multivariate binary logistic regression analysis was employed to determine the association between the MY-MINDD score (categorised into four tertiles) and MCI. This approach was completely exploratory, which allows researchers to identify the most important predictors of MCI in an effective and efficient manner and provides a way to compare different models and identify the best model for predicting MCI. Model 1 adjusted for age, education years, household income and smoking status. Model 2 incorporated the above covariates along with health conditions and functional status: IADL, GDS, hypertension, and diabetes. Model 3 included adjustments for all covariates in models 1 and 2, as well as the anthropometric parameter: BMI. Adjusted odds ratios (adj ORs) for MCI and non-MCI, along with their 95% confidence intervals (CIs), were projected after accounting for all covariates. Additionally, the Mann-Whitney U test was applied to compare the differences in each food component score of the MY-MINDD between the MCI and non-MCI groups.

## Results

### Participants characteristics

Table 3 displays the baseline attributes of the 810 participants categorised into tertiles of the diet score (tertile 1, tertile 2, tertile 3, and tertile 4). The average MY-MINDD score for all participants was 6.4 ± 0.1 (out of a total of 11 points), with a lower score observed in participants in the first tertile (4.2 ± 0.8) in comparison to the fourth tertile (8.6 ± 0.6). Significant differences were noted in age and the MY-MINDD score across all tertiles (*p* < 0.05). For BMI, there was a significant difference between tertile 1 and tertile 4 (*p* < 0.05), whereas no significant difference was observed between tertile 2 and tertile 4.

**Table 3:** Attributes of the participants according to tertiles of the MY-MINDD score (*n* = 810)

Characteristics	All (*n* = 810)	MY-MINDD score (range 0–11)				P value
		Tertile 1 (*n* = 189)	Tertile 2 (*n* = 186)	Tertile 3 (*n* = 263)	Tertile 4 (*n* = 172)	
MY-MINDD score^1^, mean ± SD	6.4 **±** 0.1	4.2 **±** 0.8	5.8 **±** 0.3	7.0 **±** 0.4	8.6 **±** 0.6	
Gender^2^, n (%)						0.670
Male	390 (48.1)	98 (51.9)	90 (48.4)	122 (46.4)	80 (46.5)	
Female	420 (51.9)	91 (48.1)	96 (51.6)	141 (53.6)	92 (53.5)	
Age^1^, mean ± SD	67.9 **±** 4.7	69.7 **±** 4.5	68.4 **±** 5.0	67.6 **±** 4.8	65.9 **±** 3.7	< 0.001
Ethnicity^2^, n (%)						0.111
Malay	532 (65.7)	129 (68.3)	133 (71.5)	165 (62.7)	105 (61.0)	
Non-Malay	278 (34.3)	60 (31.7)	53 (28.5)	98 (37.3)	67 (38.9)	
Marital status^2^, n (%)						0.194
Single	18 (2.2)	6 (3.2)	2 (1.1)	4 (1.5)	6 (3.5)	
Married	599 (74.0)	140 (74.1)	130 (69.9)	194 (73.8)	135 (78.5)	
Divorce	10 (1.2)	2 (1.1)	5 (2.7)	2 (0.8)	1 (0.6)	
Widow/Widower	183 (22.6)	41 (21.7)	49 (26.3)	63 (24.0)	30 (17.4)	
Education level^2^, n (%)						< 0.001
Primary	343 (42.3)	99 (52.4)	78 (41.9)	112 (42.6)	54 (31.4)	
Secondary	319 (39.4)	55 (29.0)	86 (46.2)	121 (46.0)	87 (50.6)	
Tertiary	67 (8.3)	3 (1.6)	7 (3.8)	12 (4.6)	15 (8.7)	
None	81 (10.0)	32 (16.9)	15 (8.1)	18 (6.8)	16 (9.3)	
Education years^1^, mean (SD)	7.7 **±** 4.2	7.5 **±** 4.0	7.7 **±** 4.2	7.5 **±** 4.1	7.9 **±** 4.4	0.386
Body mass index (kg/m^2^)^1^, mean ± SD	25.6 **±** 4.2	26.4 ± 4.8	25.4 ± 4.5	25.4 ± 3.8	25.3 ± 3.6	0.026
Body mass index^2^ (kg/m^2^), n (%)						0.005
< 18.5	22 (2.7)	4 (2.1)	8 (4.3)	7 (2.7)	3 (1.7)	
18.5–24.9	350 (43.2)	80 (42.3)	81 (43.5)	111 (42.2)	78 (45.3)	
25–29.9	336 (41.5)	65 (34.4)	78 (41.9)	113 (43.0)	80 (46.5)	
≥ 30	102 (12.6)	40 (21.2)	19 (10.2)	32 (12.2)	11 (6.4)	
Hypertension^2,3^, n (%)						0.965
Yes	302 (37.3)	70 (37.0)	67 (36.0)	101 (38.4)	64 (37.2)	
No	508 (62.7)	119 (63.0)	119 (64.0)	162 (61.6)	108 (62.8)	
Diabetes^2,3^, n (%)						0.459
Yes	163 (20.1)	42 (22.2)	30 (16.1)	56 (21.3)	35 (20.3)	
No	647 (79.9)	147 (77.8)	156 (83.9)	207 (78.7)	137 (79.7)	
Hyperlipidemia^2,3^, n (%)						0.709
Yes	256 (31.6)	64 (33.9)	62 (33.3)	80 (30.4)	50 (29.1)	
No	554 (68.4)	125 (66.1)	124 (66.7)	183 (69.6)	122 (70.9)	
Smoking^2,3^, n (%)						0.565
Yes	213 (26.3)	48 (25.4)	56 (30.1)	68 (25.9)	41 (23.8)	
No	597 (73.7)	141 (74.6)	130 (69.9)	195 (74.1)	131 (76.2)	
Social activity^2,3^, n (%)						0.008
No/seldom active	65 (8.0)	21 (11.1)	5 (2.7)	20 (7.6)	19 (11.0)	
Active	745 (92.0)	168 (88.9)	181 (97.3)	243 (92.4)	53 (89.0)	
Exercise^2,3^, n (%)						0.034
No	389 (48.0)	100 (52.9)	89 (47.8)	123 (46.8)	77 (44.8)	
Seldom	121 (14.9)	33 (17.5)	23 (12.4)	33 (12.5)	32 (18.6)	
Sometimes	49 (6.0)	15 (7.9)	7 (3.8)	16 (6.1)	11 (6.4)	
Frequent	97 (12.0)	12 (6.3)	23 (12.4)	44 (16.7)	18 (10.5)	
Very frequent	154 (19.0)	29 (15.3)	44 (23.7)	47 (17.9)	34 (19.8)	
Depressive symptoms^2^, n (%) a						0.375
No depressive symptoms	747 (92.2)	175 (92.6)	168 (90.3)	248 (94.3)	156 (90.7)	
Depressive symptoms	63 (7.8)	14 (7.4)	18 (9.7)	15 (5.7)	16 (9.3)	
Functional status^1^, mean ± SD	13.0 **±** 1.5	13.2 **±** 1.3	12.9 **±** 1.7	13.1 **±** 1.4	12.8 **±** 1.8	0.693

*Abbreviations* MY-MINDD, Malaysian version of the MIND; ^1^One-way ANOVA test; ^2^Crosstab chi-square test was used to compare distributions across the tertiles of diet score; ^3^self-reported

### Malaysian-MIND diet and cognitive impairment

Table 4 presents a comparison of various baseline characteristics between the MCI and non-MCI groups. As indicated in Table 4, the prevalence of MCI was 30.2%. The MCI group exhibited older age (*p* < 0.01), lower household income (*p* < 0.001), fewer education years (*p* < 0.001), lower skeletal muscle mass (*p* < 0.05), higher BMI (*p* < 0.05), lower IADL score (*p* < 0.001) and lower MY-MINDD score (*p* < 0.001) compared to the non-MCI group. Regarding medical history, the MCI group participants had a higher prevalence of hypertension (51.4%) and diabetes mellitus (27.3%) as against the non-MCI group (31.2% and 17.0%), respectively (*p* < 0.05). Likewise, the MCI group had a higher proportion of depressive symptoms (12.7%) in comparison to the non-MCI group (5.7%). In respect to cognitive function, the non-MCI group had a higher score of M-MMSE (*p* < 0.001), RAVLT (*p* < 0.001), Digit symbol substitution (*p* < 0.001), VR I (*p* < 0.001) and VR II (*p* < 0.001). The MY-MINDD score demonstrated significant weak correlations with MMSE (*r* = 0.154, *p* < 0.001), digit span (*r* = 0.148, *p* < 0.001), and visual reproduction I (*r* = 0.085, *p* < 0.05).

**Table 4:** Comparison of different characteristics between MCI group and non-MCI group (Express as n (%) and mean ± SD)

Components	Total	Non-MCI (*n* = 565)	MCI (*n* = 245)	P value
**Sociodemographic**				
Gender, n (%)				0.355
Male	390 (48.1)	266 (47.1)	124 (50.6)	
Female	420 (51.9)	299 (52.9)	121 (49.4)	
Age, mean ± SD	67.9 ± 4.7	67.6 ± 4.7	68.6 ± 4.7	0.003
Ethnicity, n (%)				0.202
Malay	532 (65.7)	379 (67.1)	153 (62.4)	
Non-Malay	278 (34.3)	186 (32.9)	92 (37.6)	
Marital Status, n (%)				0.211
Married	599 (74.0)	425 (75.2)	174 (71.0)	
Non-Married	211 (26.0)	140 (24.8)	71 (29.0)	
Education level, n (%)				< 0.001
Primary	343 (42.3)	204 (36.1)	139 (56.7)	
Secondary	349 (43.1)	285 (50.4)	64 (26.1)	
Tertiary	37 (4.5)	32 (5.7)	5 (2.0)	
None	81 (10.0)	44 (7.8)	37 (15.1)	
Education years, mean ± SD	7.7 ± 4.2	8.2 ± 4.1	6.4 ± 4.1	< 0.001
Household income, mean ± SD	1724.1 ± 2096.7	1908.5 ± 2293.9	1298.9 ± 1467.1	< 0.001
**Anthropometry**				
Fat percentage (%), mean ± SD	38.2 ± 10.3	38.0 ± 10.2	38.5 ± 10.5	0.384
Skeletal muscle mass, mean ± SD	25.6 ± 10.4	26.1 ± 10.6	24.5 ± 10.0	0.045
Body mass index (kg/m^2^), mean ± SD	25.6 **±** 4.2	25.4 **±** 4.0	26.1 **±** 4.7	0.048
Body mass index (kg/m^2^), n (%)				0.005
< 18.5	22 (2.7)	16 (2.8)	6 (2.4)	
18.5–24.9	350 (43.2)	246 (43.5)	104 (42.4)	
25–29.9	336 (41.5)	247 (43.7)	89 (36.3)	
≥ 30	102 (12.6)	56 (9.9)	46 (18.8)	
**Self-reported medical history**				
Hypertension^1^, n (%)				< 0.001
Yes	302 (37.3)	176 (31.2)	126 (51.4)	
No	508 (62.7)	389 (68.8)	119 (48.6)	
Diabetes^1^, n (%)				< 0.001
Yes	163 (20.1)	96 (17.0)	67 (27.3)	
No	647 (79.9)	469 (83.0)	178 (72.7)	
Hyperlipidemia^1^, n (%)				0.115
Yes	256 (31.6)	169 (29.9)	87 (35.5)	
No	554 (68.4)	396 (70.1)	158 (64.5)	
**Lifestyles**				
Smoking status^1^, n (%)				
Yes	213 (26.3)	138 (24.4)	75 (30.6)	0.066
No	597 (73.7)	427 (75.6)	170 (69.4)	
Social activity^1^, n (%)				0.706
No/seldom active	65 (8.0)	44 (7.8)	21 (8.6)	
Active	745 (92.0)	521 (92.2)	224 (91.4)	
Exercise^1^, n (%)				0.224
No	389 (48.0)	262 (46.4)	127 (51.8)	
Seldom	121 (14.9)	90 (15.9)	31 (12.7)	
Sometimes	49 (6.0)	39 (6.9)	10 (4.1)	
Frequent	97 (12.0)	71 (12.6)	26 (10.6)	
Very frequent	154 (19.0)	103 (18.2)	51 (20.8)	
**Psychosocial and functional status**				
Depressive symptoms, n (%)				< 0.001
No depressive symptoms	747 (92.2)	533 (94.3)	214 (87.3)	
Depressive symptoms	63 (7.8)	32 (5.7)	31 (12.7)	
Functional status, mean ± SD	13.0 ± 1.5	13.1 ± 1.6	12.8 ± 1.4	< 0.001
**Dietary**				
MY-MINDD score, mean ± SD	6.4 ± 1.6	6.6 ± 1.6	6.0 ± 1.7	< 0.001
**Cognitive function**				
M-MMSE	24.7 ± 3.9	25.4 ± 4.1	23.1 ± 2.9	< 0.001
Digit span	8.3 ± 2.4	8.4 ± 2.4	8.2 ± 2.4	0.144
RAVLT	4.1 ± 3.3	4.6 ± 3.5	2.9 ± 2.4	< 0.001
Digit symbol substitution	5.6 ± 2.8	6.0 ± 2.8	4.7 ± 2.4	< 0.001
VR I	25.2 ± 8.2	26.4 ± 8.2	22.4 ± 7.5	< 0.001
VR II	17.3 ± 11.7	19.3 ± 11.7	12.9 ± 10.4	< 0.001

*Abbreviations* MY-MINDD, Malaysian version of the MIND; MCI, mild cognitive impairment; M-MMSE, Malay version Mini-Mental State Examination; RAVLT, Rey Auditory Verbal Learning Test; VR, Visual reproduction; ^1^self-reported

Hierarchical multivariate logistic regression was employed to investigate the relationship between the MY-MINDD score (categorised into tertiles) and MCI, as illustrated in Table 5. The analysis was adjusted for various factors, including age, education years, household income, smoking status, functional status, depressive symptoms, hypertension, diabetes and BMI. In the fully adjusted model (Model 3), having the highest tertile (tertile 4) score was significantly associated with a lower likelihood of MCI (odds ratio (OR) = 0.43, 95% confidence interval (CI): 0.26–0.72, *p* < 0.001). Similarly, tertiles 2 and 3 also showed significant associations (OR = 0.52, 95% CI: 0.33–0.84, *p* < 0.01 and OR = 0.50, 95% CI: 0.33–0.77, *p* < 0.01, respectively) in comparison to the lowest tertile.

**Table 5:** Odds ratio (OR) and 95% confidence interval (CI) of estimated effects for tertiles of the diet score of cognitive impairment

Variables	Model 1^a^	Model 2^b^	Model 3^c^
	OR (95% CI)	OR (95% CI)	OR (95% CI)
Diet Score			
Tertile 1	1(reference)	1(reference)	1(reference)
Tertile 2	0.54(0.35,0.84) ‘	0.52(0.32,0.82) ‘	0.52(0.33,0.84) ‘
Tertile 3	0.51(0.34,0.78) *	0.49(0.32,0.76) *	0.50(0.33,0.77) *
Tertile 4	0.46(0.28,0.75) *	0.41(0.25,0.69) *	0.43(0.26,0.72) *
Age	1.03(1.00,1.07) #	1.04(1.00,1.08) #	1.04(1.00,1.08) #
Education years	0.92(0.88,0.95) *	0.94(0.90,0.98) ‘	0.93(0.89,0.98) *
Household income	1.00(1.00,1.00) #	1.00(1.00,1.00)	1.00(1.00,1.00)
Smoking status	0.92(0.78,1.10)	0.93(0.77,1.12)	0.91(0.75,1.09)
Functional status		0.98(0.88,1.09)	0.98(0.88,1.09)
Depressive symptoms		1.21(1.12,1.30) *	1.20(1.12,1.30) *
Hypertension		1.96(1.38,2.78) *	1.99(1.40,2.83) *
Diabetes		1.24(0.82,1.86)	1.19(0.79,1.80)
Body Mass Index (kg/m²)			1.22(0.97,1.52) #

Hierarchical multivariable logistics regression was used to test the association between the diet score (modelled in tertiles) and cognitive impairments; Significant at *P < 0.001; ’*P* < 0.01; #*P* < 0.05; (a) Model 1: adjusted for age, education years, household income and smoking status; (b) Model 2: adjusted for age, education years, household income, smoking status, IADL, GDS, hypertension and diabetes; (c) Model 3: adjusted for age, education years, household income, smoking status, IADL, GDS, hypertension and diabetes and body mass index

As depicted in Table 6, whole grain (*p* < 0.001), deep-sea fish (not fried) (*p* < 0.001), poultry (not fried) (*p* < 0.001), other vegetables (*p* < 0.05), and the total MY-MINDD scores (*p* < 0.001) were significantly higher in the non-MCI groups. Conversely, red meat was notably higher in the MCI group (*p* < 0.001).

**Table 6:** Comparison of each food components scores of the MY-MINDD between MCI and non-MCI groups

Components	Total (mean ± standard deviation)	Non-MCI (mean ± standard deviation)	MCI (mean ± standard deviation)	P value
Whole grains	0.12 ± 0.28	0.14 ± 0.30	0.07 ± 0.22	< 0.001
Green leafy vegetables	0.73 ± 0.39	0.74 ± 0.39	0.73 ± 0.39	0.701
Other vegetables	0.48 ± 0.43	0.50 ± 0.43	0.42 ± 0.41	0.017
Flavonoid-rich fruits	0.49 ± 0.47	0.49 ± 0.47	0.48 ± 0.47	0.694
Deep-sea fish (not fried)	0.57 ± 0.47	0.60 ± 0.47	0.50 ± 0.48	< 0.001
Legumes and Soy products	0.30 ± 0.42	0.31 ± 0.42	0.29 ± 0.41	0.699
Poultry (not fried)	0.54 ± 0.47	0.58 ± 0.46	0.44 ± 0.47	< 0.001
Desserts, sweetened *kuih* and beverages	0.77 ± 0.38	0.79 ± 0.35	0.73 ± 0.44	0.066
Butter, Margarine	0.92 ± 0.25	0.92 ± 0.25	0.92 ± 0.24	0.961
Red meat	0.91 ± 0.24	0.93 ± 0.21	0.87 ± 0.29	< 0.001
Fried/fast foods	0.59 ± 0.44	0.60 ± 0.43	0.56 ± 0.46	0.232
Total MIND diet score	6.41 ± 1.62	6.59 ± 1.56	6.01 ± 1.67	< 0.001

*Abbreviations* MY-MINDD, Malaysian version of the MIND; MCI, mild cognitive impairment

## Discussion

This study has successfully developed the MY-MINDD scores and validated it with the risk of MCI among multiethnic Malaysians older adults. In this study, adhering to the MY-MINDD diet in the highest tertile (tertile 4) was associated with a remarkable 57% reduction in the risk of MCI in the fully adjusted model. This aligns with findings from the United States, where a 53% reduction in the rate of Alzheimer’s Disease was estimated for individuals in the highest tertile of MIND scores [10]. Similarly, the highest tertile of cMIND diet score in China was linked to a 40% reduction in the risk of cognitive impairment based on a longitudinal study of the cMIND diet [7].

Several components of the MY-MINDD score, including whole grains, other vegetables, deep-sea fish, poultry, red meat, and the total MIND diet score, exhibit noteworthy relationships with MCI. Such food groups contribute positively to cognition through diverse mechanisms. In a cohort study conducted in the United States by Liu et al. (2023), a higher and more frequent consumption of whole grains was linked to a decelerated drop in global cognition, episodic memory, and perceptual speed [20]. A systematic review by Ross et al. (2023) highlighted that most studies reported a positive relationship between whole-grain consumption and measures of cognitive decline, anxiety, and mood. Furthermore, a higher intake of whole grains, in contrast to refined grains, always correlates with a reduced risk of type 2 diabetes and cardiovascular disease, both of which are factors related to cognitive decline [55]. Oats, rich in health benefits, contain dietary fibres like β-glucan, resistant to digestion and absorption in the small intestine, thereby mitigating blood cholesterol—a factor linked to a lowered risk of Alzheimer’s disease [19].

Moreover, the current research’s observations indicate a significant relationship between non-fried fish and poultry scores and MCI, aligning with the findings of Oyen et al. (2018) [56]. This correlation is consistent with the meta-analysis by Zhang et al. (2016), providing systematic evidence that increased consumption of fish and marine-derived DHA may be linked to reduced risks of dementia and Alzheimer’s disease, whereas higher total PUFA intake might be related to lower risks of Parkinson’s disease and MCI [28]. Adding to these insights, a 13-year longitudinal study in France noted that non-fried poultry consumers with an intake exceeding the median (17 g/d) had a decreased risk of cognitive decline, as observed by a systematic review by Zhang et al. (2020) [57]. A similar significant pattern emerged in the red meat score, with higher scores observed in non-MCI groups, indicating lower red meat intake in comparison to MCI groups. This observation resonates with the longitudinal study by Granic et al. (2015), which identified a higher intake of red meat/meat dishes as linked with worse global and attention-specific cognition in very elderly participants of the Newcastle 85 + research [58]. The elevated saturated fats in red meat may heighten the risk of impaired cognition by disordering peripheral and brain lipid homeostasis, impacting neuronal membrane properties, signal transduction of neurons, and synaptic plasticity, and raising the production of amyloid-beta (Aβ) – a characteristic of Alzheimer’s disease pathology [58]. Notably, the other food constituents in MY-MINDD did not exhibit significant differences between non-MCI and MCI groups, which contradicts some earlier research works [7, 19]. These disparities in findings may stem from differences in cooking techniques and dietary patterns between the Malaysian populace and other cohorts [59]. Further investigations are warranted to elucidate the precise associations or explore more appropriate classifications for green leafy vegetables, flavonoid-rich fruits, legumes and soy products, desserts, sweetened *kuih* and beverages, butter/margarine, and fried/fast foods.

This study possesses several notable strengths. Firstly, a tailored brain-healthy dietary model, MY-MINDD, was meticulously developed for the Malaysian population through thorough reviews and literature searches of prior research works. This adaptation aimed to better align with the unique dietary patterns and food cultures prevalent in Malaysia. Secondly, the utilisation of local longitudinal data from the LRGS-TUA and FRGS studies, acquired through a robust questionnaire, enhances the credibility of its relationship to MCI. The diverse samples, encompassing various provinces in Malaysia, renders it highly representative of the Malaysian older adult population, thereby bolstering the generalisability of our observations [3, 19].

Nevertheless, several limitations were found in this study. Firstly, the dietary pattern was deduced from the Dietary History Questionnaire (DHQ), introducing the possibility of under- or over-reporting of dietary intakes due to issues like memory loss or memory problems. Such factors could impact the rationality and dependability of dietary intake data. Next, our reliance on pre-existing databases to gather participant’s data for the study involving older adults was hampered by the absence of information on certain target variables that had 1% of missing data. To address this issue, multiple imputations were employed in the analysis, aiming to mitigate biased estimates by filling in missing values through the generation of plausible numbers obtained from distributions of and relationships among the observed variables in the dataset. Investigating the correlation between adherence to MY-MINDD diet and the progression of dementia presents a promising avenue for future research.

## Conclusion

Adherence to the newly developed MY-MINDD, especially at the highest tertile, demonstrated a potential 57% reduction in the risk of MCI in the full-adjusted model after accounting for covariates. However, future studies with an interventional design and the inclusion of objective measures of cognitive impairment, like neuroimaging and metabolomics approaches were suggested to gain deeper insights into the association between MY-MINDD and cognitive impairment.

## Data Availability

The datasets used and/or analysed during the current study are available from the corresponding author on reasonable request.

## Abbreviations

MY-MINDD: Malaysian-MIND diet
CI: Cognitive impairment
DASH: Dietary Approaches to Stop Hypertension
MCI: Mild cognitive impairment
LRGS-TUA: Long-term Research Grant Scheme – Towards Useful Aging
FRGS: Fundamental Research Grant Scheme
CD: Cognitive decline
CF: Cognitive function
LDL: Low-density lipoprotein
RAVLT: Rey Auditory Verbal Learning Test
IADL: Instrumental Activities of Daily Living
BMI: Body Mass Index
MMSE: Mini Mental State Examination
DHQ: Dietary History Questionnaire
GDS: Geriatric Depression Scale
